# ﻿Notes on two species of *Massuria* Thorell, 1887 (Arachnida, Araneae, Thomisidae) from China with description of a new species

**DOI:** 10.3897/zookeys.1175.105446

**Published:** 2023-08-18

**Authors:** Cong-zheng Li, Yan-bin Yao, Yong-hong Xiao, Xiang Xu, Ke-ke Liu

**Affiliations:** 1 College of Life Science, Jinggangshan University, Ji’an 343009, Jiangxi, China Jinggangshan University Ji’an China; 2 Jinshan College of Fujian Agriculture and Forestry University, Fuzhou 350007, Fujian, China Jinshan College of Fujian Agriculture and Forestry University Fuzhou China; 3 College of Life Science, Hunan Normal University, Changsha 410081, Hunan, China Hunan Normal University Changsha China

**Keywords:** Crab spiders, new combination, single sex, supplement, taxonomy

## Abstract

Two species assigned to *Massuria* Thorell, 1887 are reviewed. The female of *Massuriabandian* Tang & Li, 2010 is described for the first time from Jianfengling National Natural Reserve, Hainan Province, China. The species *Diaeasimplex* Xu, Han & Li, 2008 is described as a synonym of *Massuriabellula* Xu, Han & Li, 2008 based on female and male specimens from Guangdong Province, China. *Massuriamin***sp. nov.** described as a new species (female, Fujian Province, China). Detailed illustrations and a distribution map are provided for these three species of *Massuria*.

## ﻿Introduction

Based on the combination of morphological and molecular identification methods, approximately 750 new spider species have been reported from China in the last three years (Li 2020; Yao et al. 2021; Liu et al. 2022a, b; Lu et al. 2022). Many new taxa have been discovered, which means that more taxa need to be revised, re-assigned, or supplemented with appropriate identification methods; one such family is the crab spiders Thomisidae Sundevall, 1833. Although there are several recent publications dealing with descriptions of previously unknown sexes of Chinese crab spiders, there are still many species requiring study (Meng et al. 2019; Wang et al. 2020; Lin et al. 2022; Liu et al. 2022b; Zhong et al. 2022).

*Massuria* was established by Thorell (1887) based on a single female specimen described as *M.angulata* Thorell, 1887 from Myanmar. Lehtinen (2004) transferred two *Pistius* species recorded from India to this genus, including *M.roonwali* (Basu, 1964) and *M.sreepanchamii* (Tikader, 1962), the latter including the first description of males in this genus. The generic characters were better understood after Ono (2002) and Tang and Li (2010b) described three new species, *M.bandian* Tang & Li, 2010, *M.ovalis* Tang & Li, 2010 and *M.watari* Ono, 2002.

Currently, there are nine valid species of *Massuria* recorded from Asia (World Spider Catalog 2023). Six of these species are known from a single sex: four were described from females and two from males (World Spider Catalog 2023). Only two publications supplemented the single sex species, Tikader (1968) and Ono (2009). When we examined thomisid spider specimens collected by spider enthusiasts from Guangdong and Fujian and borrowed by Dr Guo Tang from the Institute of Zoology, Chinese Academy of Sciences, one new thomisid species was identified as new and is described in this paper, *Massuriamin* sp. nov. Further, we found that the female of *Massuriabellula* Xu, Han & Li, 2008 is a synonym of the male of *Diaeasimplex* Xu, Han & Li, 2008 and re-combined as *Massuriasimplex* (Xu, Han & Li, 2008) comb. nov.

## ﻿Materials and methods

Specimens were examined using a SZ6100 stereomicroscope. Both male and female copulatory organs were dissected and examined in 80% ethanol using an Olympus CX43 compound microscope with a KUY NICE CCD camera. Epigynes were cleared with pancreatin solution (Álvarez-Padilla and Hormiga 2007).

The measurements were taken using a stereomicroscope (AxioVision SE64 Rel. 4.8.3) and are given in millimeters. The body lengths of all specimens exclude the chelicerae and spinnerets. Terminology of the male and female genitalia follows Lin et al. (2023). Leg measurements are given as total length (femur, patella, tibia, metatarsus, tarsus). The abbreviations used in the figures and text are as follows:

**ALE** anterior lateral eye;

**AME** anterior median eye;

**ASM-JGSU** Animal Specimen Museum, College of Life Science, Jinggangshan University;

**CD** copulatory duct;

**CO** copulatory opening;

**d** dorsal;

**Em** embolus;

**FD** fertilization duct;

**IZCAS**Institute of Zoology, Chinese Academy of Sciences;

**MOA** median ocular area;

**MP** median plate;

**p** prolateral;

**PLE** posterior lateral eye;

**PME** posterior median eye;

**r** retrolateral;

**RTA** retrolateral tibial apophysis;

**Spe** spermatheca;

**v** ventral;

**VTA** ventral tibial apophysis.

## ﻿Taxonomy

### ﻿Family Thomisidae Sundevall,1833

**Genus *Massuria* Thorell, 1887**

#### ﻿ Massuria bandian

Taxon classificationAnimaliaAraneaeThomisidae

Tang & Li, 2010

33844E50-F682-5F8B-8A8D-F0A000AF13C7

Figs 1 , 2

Massuria bandian Tang & Li, 2010b: 29, figs 18A–D, 19A–B (holotype not examined).

##### Material examined.

1f, 9 m: China: Hainan Province: Ledong County, Jianfengling National Natural Reserve, Mingfenggu Scenic Spot, 18°44'25.87"N, 108°50'47.83"E, 14 August 2010, G. Zheng leg. (Tho-132, HUN).

##### Diagnosis.

**Figure 1.**
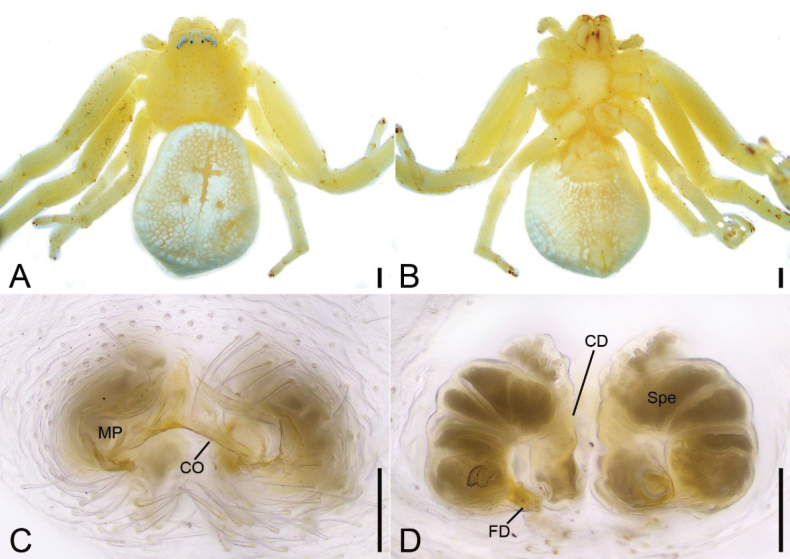
*Massuriabandian* Tang & Li, 2010, female **A** habitus, dorsal view **B** same, ventral view **C** epigyne, dorsal view **D** same, ventral view. Abbreviations: CD – copulatory duct, CO – copulatory opening, FD – fertilization duct, MP – median plate, Spe – spermatheca. Scale bars: 0.5 mm (**A, B**); 0.1 mm (**C**, **D**).

The females of this species resemble that of *Massuriamin* sp. nov. (Fig. 5A, C, D) in having a triangular median plate, but differs from it by the carapace lacking dots (vs. present), the slightly parallel copulatory ducts (vs. W-shaped) and the C-shaped spermathecae (vs. fan-shaped) (Fig. 1A, C, D). Male can be easily distinguished from other *Massuria* species (Xu et al. 2008; Tang and Li 2010b; Lin et al. 2023) by the legs I and II lacking annulations (vs. present), the retrolateral tibial apophysis lacking basal apophysis (vs. present) and the embolus arising from a 3 o’clock (vs. other positions).

##### Description.

**Female. *Habitus*** (Fig. 1A, B). Total length 5.85. Carapace length 2.37, width 2.69, with many short club-shaped setae. Eye diameters and interdistances: AME 0.05, ALE 0.09, PME 0.05, PLE 0.07; AME–AME 0.25, ALE−AME 0.18, ALE−ALE 0.67, PME–PME 0.23, PLE−PME 0.35, PLE−PLE 1.02, AME−PME 0.27, AME-PLE 0.47, ALE−PLE 0.20, MOA 0.36 long, front width 0.35, back width 0.33. Chelicerae yellow, straight, robust, with several thick setae on frontal surface, lacking promarginal and retromarginal teeth. Endites yellow, with a distinct constriction medially, anterolaterally fan-shaped. Labium oval, longer than 2/3 of endite. Sternum (Fig. 1B) yellow, broadly oval, slightly longer than wide. Legs yellow (Fig. 1A, B), without annulation, measurements (Fig. 1A, B): I 8.75 (2.8, 1.44, 2.37, 1.41, 0.73); II 9.08 (2.74, 1.51, 2.41, 1.67, 0.75); III 5.36 (1.7, 0.82, 1.3, 0.91, 0.63); IV 5.05 (1.99, 0.83, 1.27, 0.55, 0.41); setation (Fig. 1A, B): I Fe: d1, p1; Pa: d1; Ti: v8; Mt: p2, r1, v12; II Pa: d1; Pa: d1; Ti: v8; Mt: p1, r1, v12; III Ti: d1; Mt: d1; IV: Ti: d1; Mt: d1. Abdomen (Fig. 1A, B): length 3.48, width 3.22, with a yellow cross-shaped mark on anteromedial abdomen.

***Epigyne*** (Fig. 1C, D). Median plate (MP) triangular, copulatory openings (CO) located posterolateral of median plate. Copulatory ducts (CD) relatively long, as long as spermathecal length, slightly separated. Spermathecae (Spe) sac-shaped, slightly curved, with several constrictions. Fertilization ducts (FD) short and broad, directed laterally.

**Figure 2.**
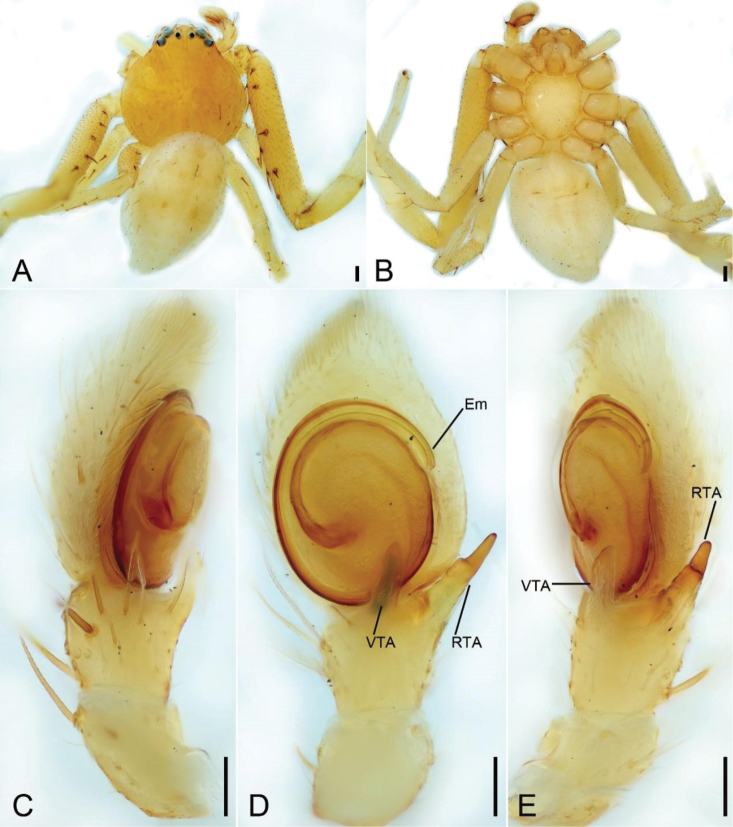
*Massuriabandian* Tang & Li, 2010, male **A** habitus, dorsal view **B** same, ventral view **C** palp, prolateral view **D** same, ventral view **E** same, retrolateral view. Abbreviations: Em – embolus, RTA – retrolateral tibial apophysis, VTA – ventral tibial apophysis. Scale bars: 0.2 mm (**A, B**); 0.1 mm (**C, D**).

**Male. *Habitus*** (Fig. 2A, B). As in female except as follows. Total length 3.53. Carapace (Fig. 2A) length 1.56, width 1.73, with several erect club-shaped setae. Eye (Fig. 2A) diameters and interdistances: AME 0.06, ALE 0.10, PME 0.05, PLE 0.09; AME–AME 0.16, AME−ALE 0.11, ALE−ALE 0.47, PME–PME 0.14, PME−PLE 0.25, PLE−PLE 0.74, ALE−PLE 0.16, AME−PME 0.18. MOA 0.29 long, front width 0.26, back width 0.26. Chelicerae yellow, straight, robust, with abundant thick setae on frontal surface, lacking promarginal and retromarginal teeth. Endites yellow, with a distinct constriction medially. Labium trapezoidal. Legs yellow (Fig. 2A, B), legs I and II without annulations on patellae, tibiae, metatarsi, and tarsi; measurements: I 8.86 (2.7, 0.84, 2.25, 1.96, 1.11); II 7.67 (1.9, 0.82, 2.3, 1.84, 0.81); III 3.2 (0.95, 0.44, 0.82, 0.56, 0.43); IV 3.44 (1.12, 0.54, 0.93, 0.45, 0.4); setation (Fig. 2A, B): I Ti: d2, p1, r2, v5; Mt: p1, r2, v8; II Fe: d5; Ti: d3, p2, r1, v4; Mt: v10; III Fe: d3; Pa: d1; Ti: d3; IV: Fe: d2; Pa: d1, r1; Ti: d4; Mt: p1, r1. Abdomen (Fig. 2A, B) oval, 1.97 long, 1.19 wide, yellowish to yellow.

***Palp*** (Fig. 2C−E). Ventral tibial apophysis (VTA) slightly shorter than tibia, directed anteriorly. Retrolateral tibial apophysis (RTA) longer than tibia, sub-medial part sharply pointed, with a thick spine-like tip. Embolus (Em) arising from 6 o’clock and ending at about 2 o’clock.

##### Distribution.

Known from Yunnan and Hainan (this article), China (Fig. 7).

#### ﻿ Massuria simplex

Taxon classificationAnimaliaAraneaeThomisidae

(Xu, Han & Li, 2008) comb. nov.

CA5C0685-93C1-5A72-8C4C-E1F3A66EC26B

Figs 3 , 4 , 6A, B

Pistius gangulyi Yaginuma & Wen, 1983: 193, fig. 1A−C (♀, misidentified).
Diaea simplex Xu, Han & Li, 2008: 14, fig. 1a−e (male holotype not examined). syn. nov.; Tang et al. 2010a (♂).
Massuria bellula Xu, Han & Li, 2008: 15, fig. 2a−c (female holotype not examined).

##### Material examined.

1f, 1 m: China: Guangdong Province: Maoming City, Gaozhou City, Changpo Town, 22°4'43.34"N, 111°6'30.51"E, 6.II.2022, Y.H. Zhong leg. (Tho-297, ASM-JGSU).

##### Diagnosis.

**Figure 3.**
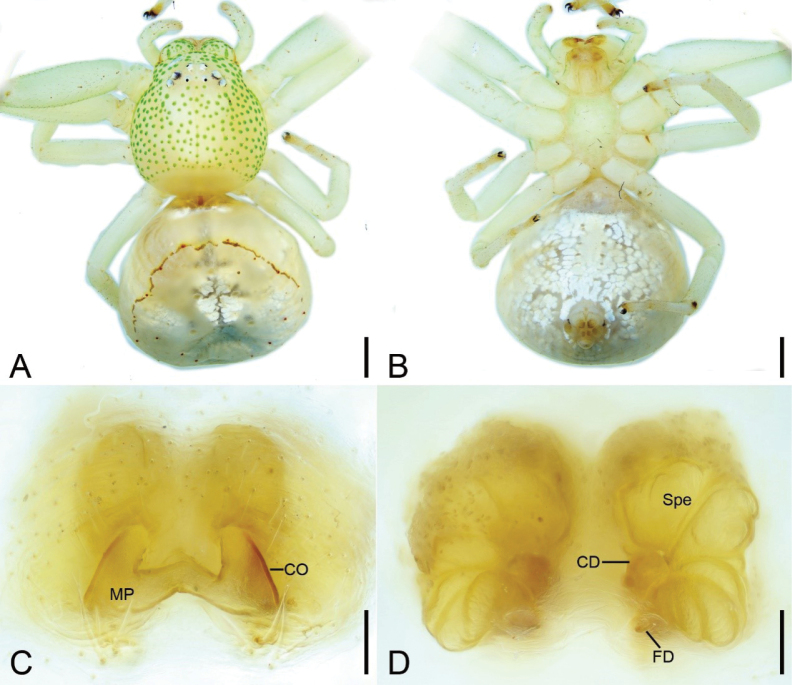
*Massuriasimplex* (Xu, Han & Li, 2008) comb. nov., female **A** habitus, dorsal view **B** same, ventral view **C** epigyne, dorsal view **D** same, ventral view. Abbreviations: CD – copulatory duct, CO – copulatory opening, FD – fertilization duct, MP – median plate, Spe – spermatheca. Scale bars: 1 mm (**A, B**); 0.1 mm (**C, D**).

**Figure 4.**
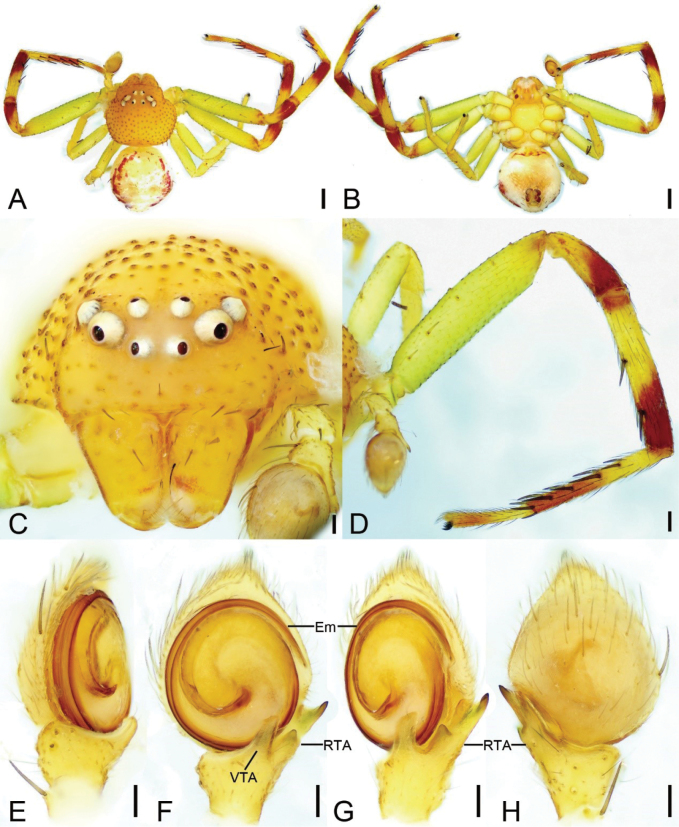
*Massuriasimplex* (Xu, Han & Li, 2008) comb. nov., male **A** habitus, dorsal view **B** same, ventral view **C** carapace, frontal view **D** left leg I, prolateral view **E** palp, ventro-prolateral view **F** same, ventral view **G** same, ventro-retrolateral view **H** same, dorsal view. Abbreviations: Em – embolus, RTA – retrolateral tibial apophysis, VTA – ventral tibial apophysis. Scale bars: 0.5 mm (**A, B**); 0.2 mm (**D**); 0.1 mm (**C, E–H**).

Female resembles those of *M.daizong* Lin & Li, 2023 (see Lin et al. 2023: 74, figs 66A, B, 67B) in having the sac-shaped spermathecae with many constrictions, but can be easily differentiated from it by the dorsal abdomen without a distinct marking (vs. a red face mask-like marking present in *M.daizong*), the W-shaped epigynal plate (vs. M-shaped in *M.daizong*), and the long longitudinal copulatory ducts (vs. short in *M.daizong*) (Fig. 3C, D). Male is very similar to that of *Massuriaovalis* Tang & Li, 2010 (see Tang and Li 2010b: 29, fig. 21A, B) in having a long embolus (Em) arising and ending at the 3 o’clock position of the tegulum, but can be recognized by the straight ventral tibial apophysis (VTA) (vs. slightly curved in *M.ovalis*) and the retrolateral tibial apophysis (RTA) with a ridge-like apophysis near the base (vs. lacking basal apophysis in *M.ovalis*) and thick spine-like apex (vs. very blunt in *M.ovalis*) in ventral view (Fig. 4G, H).

##### Description.

**Female. *Habitus*** (Figs 3A, B, 6A, B). Total length 8.07. Carapace length 3.72, width 3.52. Eye diameters and interdistances: AME 0.09, ALE 0.11, PME 0.07, PLE 0.09; AME–AME 0.41, ALE−AME 0.30, ALE−ALE 1.06, PME–PME 0.40, PLE−PME 0.50, PLE−PLE 1.48, AME−PME 0.40, AME-PLE 0.70, ALE−PLE 0.29, MOA 0.54 long, front width 0.53, back width 0.54. Leg without annulation, measurements (Fig. 3A, B): I 11.6 (3.43, 1.76, 2.87, 2.32, 1.22); II 11.79 (3.65, 1.76, 2.8, 2.37, 1.21); III 5.95 (2.17, 0.79, 1.37, 1.04, 0.58); IV 6.46 (2.3, 0.69, 1.4, 1.34, 0.73); setation (Fig. 3A, B): I Fe: d4; Ti: v8; Mt: p3, r1, v16; II Pa: d1; Ti: v5; Mt: p2, r1, v13; III Ti: d3; Mt: d3, p2; IV: Ti: d3. Abdomen (Fig. 3A, B): length 4.35, width 4.98, with a brown linear mark on anteromedial abdomen.

***Epigyne*** (Fig. 3C, D). Median plate (MP) W-shaped, copulatory openings (CO) located laterally. Copulatory ducts (CD) very short, as long as 1/3 of spermathecal length. Spermathecae (Spe) sac-shaped, median thinner than anterior and posterior parts, with several constrictions. Fertilization ducts (FD) short and broad, directed laterally.

**Male. *Habitus*** (Fig. 4A−C). Total length 3.45. Carapace (Fig. 4A) broadly oval, length 1.70, width 1.82, with densely granulated trichopores. Eye (Fig. 4C) diameters and interdistances: AME 0.07, ALE 0.09, PME 0.05, PLE 0.07; AME–AME 0.22, AME−ALE 0.15, ALE−ALE 0.61, PME–PME 0.22, PME−PLE 0.29, PLE−PLE 0.88, ALE−PLE 0.19, AME−PME 0.20. MOA 0.31 long, front width 0.31, back width 0.33. Chelicerae yellow, straight, robust, with abundant thick setae on frontal surface, lacking promarginal and retromarginal teeth. Endites yellow, with a distinct constriction medially. Labium oval, as long as 2/3 of endite. Sternum (Fig. 4B) yellow, broadly oval, wider than long. Legs yellow (Fig. 4A, B), legs I and II with brown annulations on patellae, tibiae, metatarsi, and tarsi; measurements: I 6.68 (2.04, 0.84, 1.58, 1.38, 0.84); II 6.99 (2.06, 0.87, 1.7, 1.48, 0.88); III 3.52 (0.97, 0.54, 0.88, 0.69, 0.44); IV 3.13 (0.9, 0.59, 0.82, 0.5, 0.32); setation (Fig. 4A, B): I Fe: d3, p4; Ti: d1, v4; Mt: p1, r1, v10; II Fe: d5; Ti: d1, v4; Mt: p2, r2, v6; III Fe: d2; Pa: d2; Ti: d1; IV: Fe: d2; Pa: d2, r1; Ti: d4, r1. Abdomen (Fig. 4A, B) ovoid, 1.75 long, 1.72 wide, yellow, laterally with arc-shaped filiform mark.

***Palp*** (Fig. 4E−H). Ventral tibial apophysis (VTA) slightly shorter than tibia, directed retrolaterally. Retrolateral tibial apophysis (RTA) longer than tibia, with a ridge-like apophysis and a thick spine-like tip. Embolus (Em) arising from 3 o’clock and ending at the same position.

##### Comments.

It is noteworthy that the figure of the female of *Pistiusgangulyi* presented by Yaginuma and Wen (1983) agreed well with the specimens known from Guangdong, although they had a female specimen from Hainan as the same as the male records by Tang and Li (2010a). While the holotype female of *Massuriabellula* Xu, Han & Li, 2008 was collected from the Tai Lung Experimental Station, Hong Kong, China by Ping-wing Chan on 30 June 1999. The male of *Diaeasimplex* Xu, Han & Li, 2008 was also discovered by him a week later from the same locality. The latter is the same species as the first because it has all of the diagnostic features of *Massuria*: the pentagonal abdomen with distinct submarginal pattern and the male palpal RTA modified into a distal process (Sen et al. 2015). Further examination of the male and female genitalia in this study confirms its synonymy with *Diaeasimplex* Xu, Han & Li, 2008 (compare Fig. 1 with Xu et al. 2008: 14, fig. 1) and the records of *Pistiusgangulyi* from Hainan in Yaginuma and Wen (1983) were misidentified. The species *M.bellula* should thus be regarded a synonym of *D.simplex* Xu, Han & Li, 2008.

##### Distribution.

Known only from Guangdong, Hainan (Yaginuma and Wen 1983; Tang and Li 2010a) and Hong Kong (Xu et al. 2008), China (Fig. 7).

#### ﻿ Massuria min

Taxon classificationAnimaliaAraneaeThomisidae

Yao & Li sp. nov.

537EC034-8CF3-5510-845B-0E4838EEA626

https://zoobank.org/38CB3F25-A09E-498A-B74E-BE54281F3CBE

Figs 5 , 6C, D

##### Type material.

***Holotype***: 1f: China: Fujian Province: Longyan City, Xinluo District, Jiangshan Town, 25°8'20.68"N, 116°58'53.56"E, 307 m, 1.X.2022, L.F. Wei. leg. (Tho-298, ASM-JGSU).

##### Etymology.

The specific name refers to the Chinese abbreviation for Fujian Province; noun in apposition.

##### Diagnosis.

**Figure 5.**
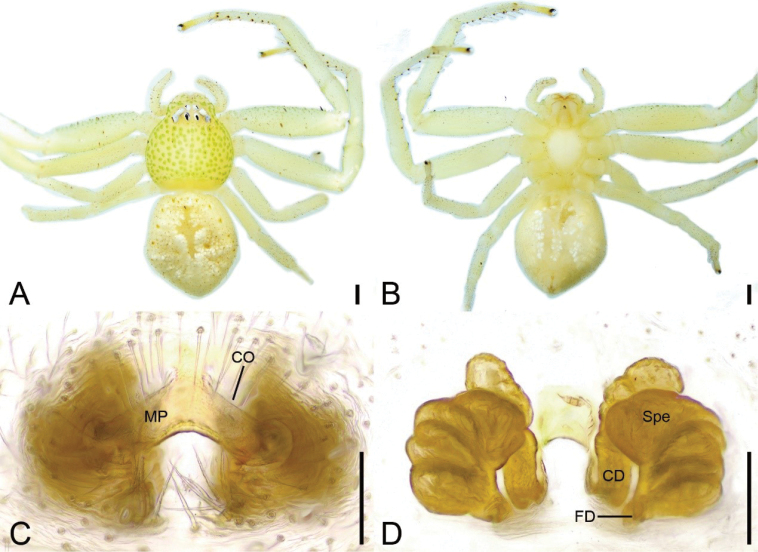
*Massuriamin* sp. nov., female holotype. **A** habitus, dorsal view **B** same, ventral view **C** epigyne, dorsal view **D** same, ventral view. Abbreviations: CD – copulatory duct, CO – copulatory opening, FD – fertilization duct, MP – median plate, Spe – spermatheca. Scale bars: 0.5 mm (**A, B**); 0.1 mm (**C, D**).

*Massuriamin* sp. nov. is similar to *M.bandian* Tang & Li, 2010 (Fig. 1A, C, D) and *M.uthoracica* Sen, Saha & Raychaudhuri, 2012 (see Sen et al. 2015: 64, figs 334, 337, 338) in having a triangular epigynal plate, but differs from it by the carapace with abundant spots (vs. absent in *M.bandian* and *M.uthoracica*), the long W-shaped copulatory ducts combined with a dorsal hood (vs. triangular in *M.bandian*; absent in *M.uthoracica*) and the fan-shaped spermathecae (vs. C-shaped in *M.bandian*; straight in *M.uthoracica*) (Fig. 5C, D).

##### Description.

**Figure 6.**
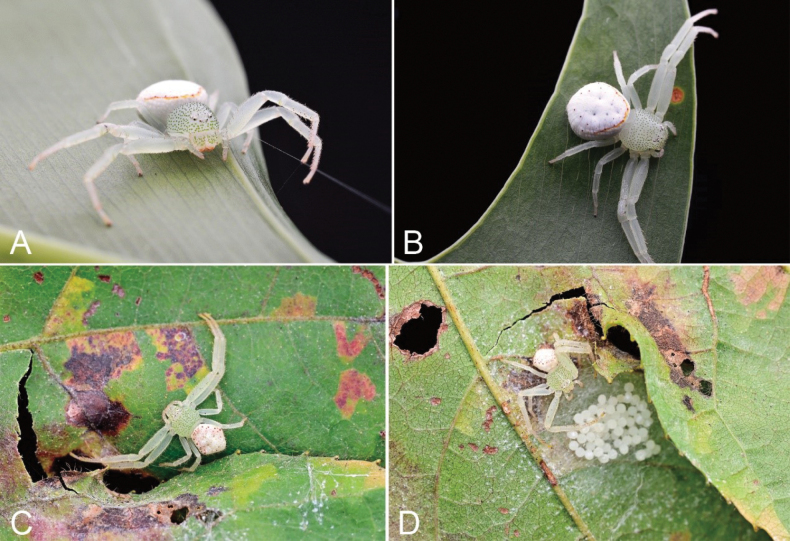
Photographs of living specimens from China **A, B***Massuriasimplex* (Xu, Han & Li, 2008) comb. nov., female **C, D***Massuriamin* sp. nov., female.

**Female. *Habitus*** (Figs 5A, B, 6C, D). Total length 5.31. Carapace (Fig. 5A) with green spots, length 2.24, width 2.30. Eyes (Fig. 5A) diameters and interdistances: AME 0.06, ALE 0.09, PME 0.05, PLE 0.09; AME–AME 0.25, ALE−AME 0.16, ALE−ALE 0.63, PME–PME 0.23, PLE−PME 0.33, PLE−PLE 0.96, AME−PME 0.25, ALE−PLE 0.22. MOA 0.34 long, front width 0.33, back width 0.33. Chelicerae yellow, straight, robust, without retromarginal or promarginal teeth. Endites yellow, medially with distinct constriction. Labium yellow, inverted U-shaped, as long as 2/3 of endite. Sternum pale to yellow, longer than wide. Legs yellow (Fig. 5A, B); measurements: I 8.15 (2.23, 1.14, 2.18, 1.8, 0.8); II 8.42 (2.23, 1.18, 2.25, 1.88, 0.88); III 4.88 (1.52, 0.83, 1.05, 0.95, 0.53); IV 5.06 (1.78, 0.71, 1.03, 0.96, 0.58); setation (Fig. 5A, B): I Fe: d4; Pa: d1; Ti: d2, v8; Mt: d1, p2, v12; II Fe: d1; Pa: d1; Ti: d1, v8; Mt: d1, p2, v11; III Pa: d1; Ti: v1; IV: Pa: d1. Abdomen (Fig. 5A, B) 3.07 long, 2.47 wide, ovoid, silver with yellow cross-shaped marks dorsally, and several yellow dots located anterolaterally.

***Epigyne*** (Figs 5C, D). Median plate (MP) triangular, copulatory openings (CO) located at antero-lateral part. Copulatory ducts (CD) W-shaped, extending from median to posterior of vulva and turn back to forward. Spermathecae (Spe) fan-shaped, with several constrictions, widely separated as long as their length. Fertilization ducts (FD) short, directed anteromedially.

**Male.** Unknown.

##### Comments.

At present, *M.ovalis* Tang & Li, 2010 is known only from the male in mainland China; therefore, the new species may be conspecific with this species.

##### Distribution.

**Figure 7.**
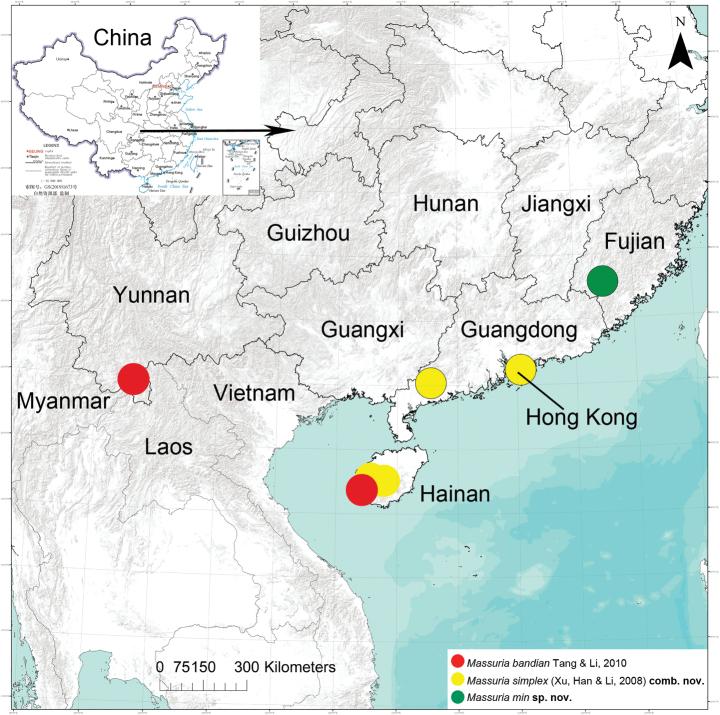
Location records of *Massuriabandian* Tang & Li, 2010, *Massuriasimplex* (Xu, Han & Li, 2008) comb. nov. and *M.min* sp. nov. from China.

Known only from the type locality (Fig. 7).

## Supplementary Material

XML Treatment for Massuria bandian

XML Treatment for Massuria simplex

XML Treatment for Massuria min

## ﻿Additional information

### Author ORCIDs

Cong-zheng Li https://orcid.org/0000-0003-3849-8433

Yan-bin Yao https://orcid.org/0000-0002-2560-9299

Yong-hong Xiao https://orcid.org/0000-0002-8074-9366

Xiang Xu https://orcid.org/0000-0001-9485-5373

Ke-ke Liu https://orcid.org/0000-0001-7822-3667

### Data availability

All of the data that support the findings of this study are available in the main text.
